# Predisposition for and Prevention of Subjective Tinnitus Development

**DOI:** 10.1371/journal.pone.0044519

**Published:** 2012-10-02

**Authors:** Sönke Ahlf, Konstantin Tziridis, Sabine Korn, Ilona Strohmeyer, Holger Schulze

**Affiliations:** Experimental Otolaryngology, University of Erlangen-Nuremberg, Erlangen, Germany; Stanford University School of Medicine, United States of America

## Abstract

Dysfunction of the inner ear as caused by presbyacusis, injuries or noise traumata may result in subjective tinnitus, but not everyone suffering from one of these diseases develops a tinnitus percept and vice versa. The reasons for these individual differences are still unclear and may explain why different treatments of the disease are beneficial for some patients but not for others. Here we for the first time compare behavioral and neurophysiological data from hearing impaired Mongolian gerbils with (T) and without (NT) a tinnitus percept that may elucidate why some specimen do develop subjective tinnitus after noise trauma while others do not. Although noise trauma induced a similar permanent hearing loss in all animals, tinnitus did develop only in about three quarters of these animals. NT animals showed higher overall cortical and auditory brainstem activity *before* noise trauma compared to T animals; that is, animals with low overall neuronal activity in the auditory system seem to be prone to develop tinnitus after noise trauma. Furthermore, T animals showed increased activity of cortical neurons representing the tinnitus frequencies *after* acoustic trauma, whereas NT animals exhibited an activity decrease at moderate sound intensities by that time. Spontaneous activity was generally increased in T but decreased in NT animals. Plastic changes of tonotopic organization were transient, only seen in T animals and vanished by the time the tinnitus percept became chronic. We propose a model for tinnitus prevention that points to a global inhibitory mechanism in auditory cortex that may prevent tinnitus genesis in animals with high overall activity in the auditory system, whereas this mechanism seems not potent enough for tinnitus prevention in animals with low overall activity.

## Introduction

Diseases of the inner ear leading to hearing loss (HL) may result in subjective tinnitus [1]. Enigmatically, not everyone suffering from HL develops a tinnitus percept and conversely not in everyone who suffers from tinnitus a permanent hearing impairment can be detected [2]. The reasons for these individual differences are still unclear and may explain why different treatments of the disease are beneficial for some patients but not for others [3].

The development of a tinnitus percept is often related to neuronal plasticity on multiple levels of the central auditory system including the auditory cortex ([4], [5], [6], [7] for review see [8]). Current models of tinnitus genesis usually consider damage of cochlear hair cells that induce an imbalance in lateral inhibition on subsequent neuronal levels as causal for such central plasticity [9], but even if the inducing event is identical not every animal or human subsequently suffers from tinnitus. In this report we follow the hypothesis that there must be some predisposition in the central auditory system of some but not all individuals that protects them from the development of subjective tinnitus. In search of this predisposition we recorded neuronal activity from the auditory brainstem and cortex of the same individuals of Mongolian gerbils before and after an acoustic trauma and compared the data obtained from animals that showed an acute tinnitus percept in behavioral testing (group T) with data from those that did not (group NT). Possible differences in these two groups of animals may further elucidate the neuronal mechanisms that lead to subjective tinnitus and thereby may help to find a prophylaxis against tinnitus development and improve actual treatments for tinnitus patients (e.g. [10]).

## Materials and Methods

### Ethics Statement

For the welfare of the animals the researchers were responsible. The gerbils were housed in a standard animal rack (Bio A.S. Vent Light, Ehret Labor- und Pharmatechnik, Emmendingen, Germany) in groups of 2 to 3 animals per cage with free access to water and food at 20 to 24°C room temperature. The use and care of animals was approved by the state of Bavaria (Regierungspräsidium Mittelfranken, Ansbach, Germany).

### Behavioral Measurements

A total of thirty five 8 to 10 weeks old male Mongolian gerbils (*Meriones unguiculatus*) purchased by Charles River (Charles River GmbH, Sulzfeld, Germany) were used in this study. Animals were handled before the beginning of the experiments and accustomed to the setup environment to minimize stress. Behavioral testing of animals was performed in an IAC (Industrial Acoustics Company GmbH, Niederkrüchten, Germany) acoustic chamber on a TMC (Technical Manufacturing Corporation, Peabody, MA, USA) low-vibration table. The behavioral setup consisted of a 15 cm long transparent acrylic tube (inner diameter 4.0 to 4.3 cm, depending on the body size of the animal) placed 10 cm in front of a speaker (Canton Plus X Series 2; Canton, Weilrod, Germany) onto a Honeywell FSG15N1A piezo sensor (Honeywell AG, Offenbach, Germany). The tube’s front end was closed with a stainless steel grate (wire mesh width 0.5 mm) allowing acoustic stimulation with no detectable distortion (signal to noise ratio at least 70 dB, checked via HP spectrum analyzer: 3563A Control Systems Analyzer; Hewlett-Packard GmbH, Böblingen, Germany). Sound pressure level was controlled via a B&K Type 2610 measuring amplifier fed with a B&K Type 2669 preamplifier/B&K Type 4190 condensor microphone combination (Brüel & Kjaer GmbH, Bremen, Germany). Stimulus generation and data acquisition was controlled using custom-made Matlab 2008 programs (MathWorks, Natick, MA, USA; stimulation/recording sampling rate 20 kHz). For sound generation the frequency response function of the speaker was calibrated to produce an output spectrum that was flat within +/−1 dB.

**Figure 1:**
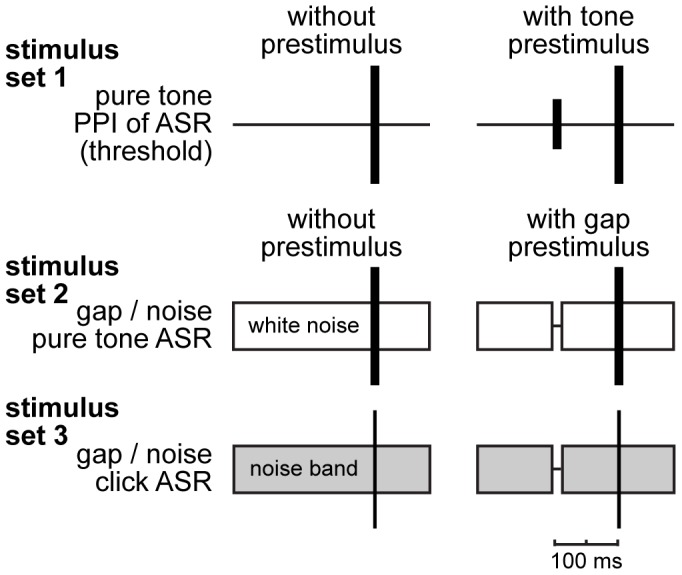
Behavioral paradigms using prepulse inhibition (PPI) of the acoustic startle response (ASR). Upper panel: Hearing threshold estimation paradigm in silence, with 90 dB pure tone startle stimuli ranging from 0.5 to 16 kHz in octave steps and prestimuli of the same frequency with intensities ranging from 0 to 50 dB SPL in 10 dB steps ( = stimulus set 1). **Center panel:** Behavioral tinnitus test (stimulus set 2, [12]) with white noise background and pure tone startle stimuli ranging from 1 to 8 kHz (in octave steps) utilizing a silent gap of 15 ms as prestimulus. **Lower panel:** Behavioral tinnitus test (stimulus set 3, [15]) with bandpass noise (width of 1 octave) of different center frequencies (1 to 16 kHz in octave steps), a click as startle stimulus and a gap as prestimulus.

Animals were placed in the tube, in which it fits well and is able to move back and forth roughly 2 cm. We allowed 15 min habituation time before 3 different types of prepulse inhibition (PPI) modulated auditory startle response (ASR) paradigms (Figure 1) were performed, usually in a single 4 h block before and 5 to 11 days (median 7 days) after the acoustic trauma. Between different stimulus sets a break of 5 minutes was used to prevent behavioral adaptation. Interstimulus intervals were varied randomly (mean 10±2.5 sec SD) and each stimulus was repeated 15 times. Stimulation paradigms were: first, an hearing threshold estimation paradigm to obtain behavioral audiograms (set 1) [11] consisting of a 90 dB SPL pure tone startle stimulus (duration: 6 ms, with 2 ms cosine-squared rise and fall ramps; 0.5 to 8.0 kHz in octave steps) preceded by 100 ms by a 0 to 50 dB SPL prestimulus of the same frequency and length. Stimuli with different frequencies were presented pseudorandomized in blocks of different prestimulus intensities starting from 0 dB SPL and rising to 50 dB SPL (cf. Figure 1, upper panel). Second, two different forms of gap-noise paradigms were used to demonstrate the existence of possible tinnitus percepts and to give a rough estimate of the perceived tinnitus frequencies [12]. The first of these used a gap-noise paradigm with a white noise background of 50 dB SPL and 90 dB SPL pure tone startle stimuli of 1 to 8 kHz in octave steps with or without a 15 ms prestimulus-gap in the noise 100 ms before the stimulus (stimulus set 2, Figure 1, center panel) with the rationale of utilizing tinnitus related anxiety as frequency specific factor that induces freezing behavior (cf. [13], [14], [15]). Each frequency was presented separately with and without gap, starting from 1 kHz in ascending steps to 8 kHz. The last paradigm (stimulus set 3) had an identical timing and presentation regime as stimulus set 2 with the startle-stimulus being a click and the noise being bandpass filtered to mean frequencies ranging from 1 to 16 kHz in octave steps and a width of ±0.5 octave (Figure 1, lower panel) with the rationale of the tinnitus frequency masking the gap in the noise band that fits its spectrum best [12]. Both gap-noise paradigms for the detection of tinnitus percepts yielded similar results (cf. below).

Data of all PPI paradigms were checked by eye: Trials in which the animals moved within 100 ms before the startle stimulus were discarded; only responses within the first 50 ms after startle stimulus onset were used for further analysis. Response data in the threshold paradigm (stimulus set 1) were fitted with a sigmoidal Boltzmann function; hearing thresholds were defined as the sound level at the inflection point of the function at each frequency before and after trauma (cf. [16]). The data in the two gap-noise paradigms (sets 2 and 3) were normalized to minimize variance of the response amplitudes and to avoid possible effects of the acoustic trauma on different stimulation frequencies. In other words, we tried to control for differences in the startle amplitudes resulting from the hearing loss at the trauma frequency. This normalization also guarantees that the reduced ASR response after acoustic trauma is not due to hearing loss rather than a tinnitus percept. The normalization was performed by dividing the actual amplitude by the median amplitude of the startle stimulus alone (set 1 pure tone startle stimulus without prestimulus). This was done for pre and post trauma conditions and all frequencies separately; then the effect of the PPI relative to pre trauma values was calculated in percent.

### Acoustic Trauma and Auditory Brainstem Recordings

An acoustic trauma at 2 kHz (115 dB SPL, 75 min) in deep ketamine-xylazine-anesthesia (mixture of ketamine, xylazine, NaCl and atropine at a mixing ratio of 9∶1∶8∶2, initial dose: 0.3 ml s.c.) was used to induce a frequency specific HL in all animals and possibly the subsequent development of a tinnitus percept. The tone was generated by a HP 33120A waveform generator, amplified and presented free-field to both ears by a speaker placed 5 cm in front of the animal’s head (Canton Plus X Series 2). Anesthesia during trauma was maintained via subcutaneous infusion of the anesthetic solution supported by a syringe pump at a rate of 0.2 to 0.3 ml/h. The animal’s body temperature was kept constant by a warming pad.

Auditory brainstem responses (ABR) were measured after the pre trauma ASR experiments but before surgery (cf. below) and again directly after the acoustic trauma to measure the acute trauma effect. Data were obtained via subcutaneously placed thin silver wire electrodes (0.25 mm diameter) using a Plexon Multichannel Acquisition Processor (Plexon system with HLK2-card; Plexon Inc., Dallas, TX, USA) after amplification by a JHM NeuroAmp 401 (J. Helbig Messtechnik, Mainaschaff, Germany) and stored with a custom-made Matlab program (10 kHz sampling rate). Auditory stimuli were generated by a custom-made Matlab program and presented free field to one ear at a time via a frequency response function corrected speaker (SinusLive neo 25S, pro hifi, Kaltenkirchen, Germany) at circa 0.5 cm distance from the animal’s pinna while the contralateral ear was tamped with an ear plug (e.g. [17]). Stimuli presented were clicks (0.1 ms duration) and pure tones (4 ms duration including 1 ms cosine-squared rise and fall times) ranging from 0.5 to 16.0 kHz in half-octave steps. 120 stimuli were presented in pairs of two phase inverted stimuli (intrastimulus interval 100 ms) and an interstimulus interval of 500 ms between stimulus pairs. Stimulation was pseudo-randomized using a fixed list of all combinations of stimulus frequencies and sound pressure levels (0 to 90 dB SPL in 5 dB steps). To obtain ABR-based audiograms the mean ABR waves were compared to the mean amplitude 200 to 100 ms before the stimulus (baseline). Thresholds were defined automatically by a custom-made Matlab program at the highest attenuation at which the evoked amplitude raised over 2 standard deviations of the baseline; data were discarded at frequencies where this procedure was not possible, e.g., at low signal to noise ratios. For additional analysis the root mean square (RMS) value of the ABR signal was calculated from 1 ms to 5 ms after stimulus onset.

### Electrophysiological Unit Recordings in Primary Auditory Cortex (AI)

Recording in AI was chosen, as it is the first and – at least for the gerbil – most important cortical representation of perceived sounds. Two to five days after obtaining baseline ASR and ABR data, i.e., before the acoustic trauma, the skull of the anesthetized animals was trepanned to expose the left auditory cortex. A 2.5 cm aluminum head-post and recording chamber was implanted. Recording began two to four days after surgery. Animals were again ketamine-xylazine anesthetized, placed on a warming pad and fixated via the aluminum head-post. Over 2 to 3 sessions every second day, single and multi-unit responses to tones in 5 to 7 tracks with 2 to 4 recording locations each in AI were recorded using tungsten microelectrodes (1 MΩ impedance, 1–2 µm tip diameter, Plexon microelectrodes PLX-ME-W-3-PC-3-1.0-A-254). Verification of recording sites was done using neuronal response characteristics (latency, tuning sharpness (Q30), temporal response patterns (phasic/tonic), tonotopic organization (cf. [18])).

Stimulation consisted of pure tones (200 ms including 1 ms cosine-squared rise and fall times) ranging from 0.25 to 16.0 kHz in quarter-octave or half-octave steps presented at 70 dB SPL with 500 ms interstimulus intervals. Additionally to these iso-intensity measurements, tuning curves were recorded using pure tones in the mentioned frequency range but at different intensities ranging from 0 to 90 dB SPL. The recorded unit activity was analyzed with custom-made Matlab and IDL programs. Best frequency (BF; frequency with highest discharge rate at 70 dB SPL) as well as spontaneous rate (mean activity within a time window from 50 ms before to stimulus onset), evoked rate at BF and evoked rates at all tested stimulation intensities and frequencies were calculated for each unit individually.

## Results

### Assessment of Noise Trauma Induced Hearing Loss and Tinnitus Development

**Figure 2:**
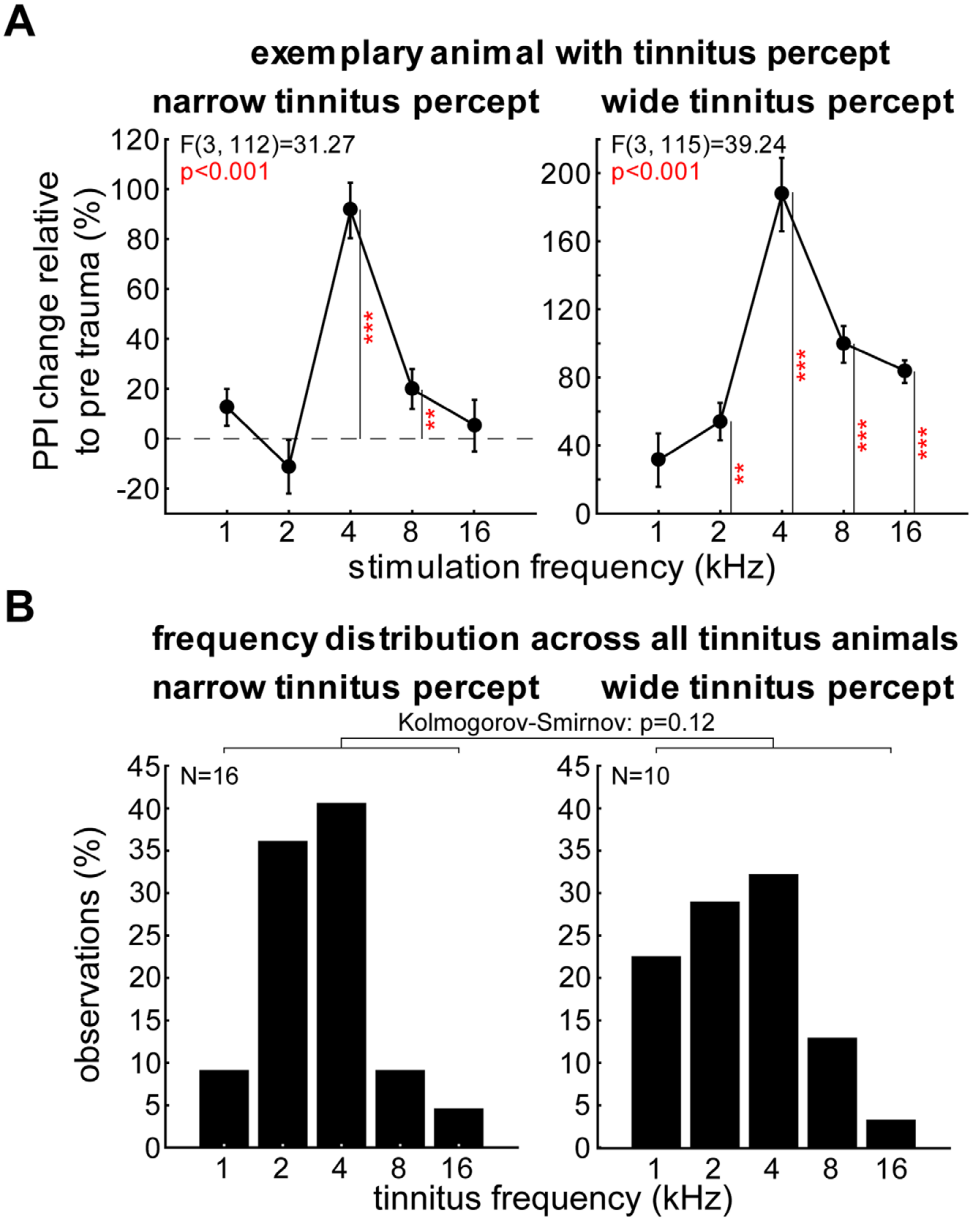
Frequency distributions of perceived tinnitus frequencies. A Data from two exemplary animals tested with stimulus set 3 with narrow (left panel) and wide tinnitus percepts (right panel), respectively. In both cases the one-factorial ANOVAs indicated frequency dependency of the PPI changes relative to pre trauma but in the first case only for two and in the second case for four frequencies. Whiskers indicate standard error of the mean (SEM) **B** Frequency distributions for narrow (16 animals) and wide tinnitus percepts (10 animals) with observation frequency given in percent. Both distributions are not significantly different from each other.

Under deep ketamine-xylazine anesthesia a total of 35 animals received a noise trauma (2 kHz pure tone, cf. Methods) that produced a spectrally defined acute HL in all animals that could be detected at least until the end of our experiments (4 animals exemplarily retested up to 16 weeks after the trauma). HL was quantified by both ABR and behavioral audiograms before and after the trauma (cf. below). Animals were also tested for tinnitus with behavioral tests (cf. Methods and [19], [20]). We determined whether an animal developed a tinnitus percept and if so approximated the individually perceived frequency. It turned out that about three quarters (26 out of 35) of the animals developed a tinnitus percept while the remaining 9 animals did not. This percept was usually strongest at frequencies at and/or 1 octave above the trauma frequency in one or both gap-noise paradigms. Figure 2A depicts the behavioral responses to stimulus set 3 of two exemplary animals with a spectrally narrow (left) or wide (right) tinnitus percept, respectively. Narrow tinnitus percepts (16/26, 61.5%) were defined as having only one or two neighboring frequencies affected, while wide tinnitus percepts had at least three frequencies affected. The example of an animal with a spectrally narrow tinnitus percept shown in the left panel of Figure 2A had a significant PPI impairment (as reflected in increased ASR) at 4 and 8 kHz only (Tukey post-hoc tests after one-factorial ANOVA). In contrast, for the animal with a spectrally wide tinnitus percept shown on the right we detected significantly impaired PPI at 4 frequencies. Figure 2B gives an overview across the frequency distributions of the tinnitus percepts in both groups. The two distributions are not significantly different from each other (Kolmogorov-Smirnov test, p = 0.12) with both showing the peak of the distributions around 2 to 4 kHz. For all further analysis we therefore combined the data of these two groups again.

**Figure 3:**
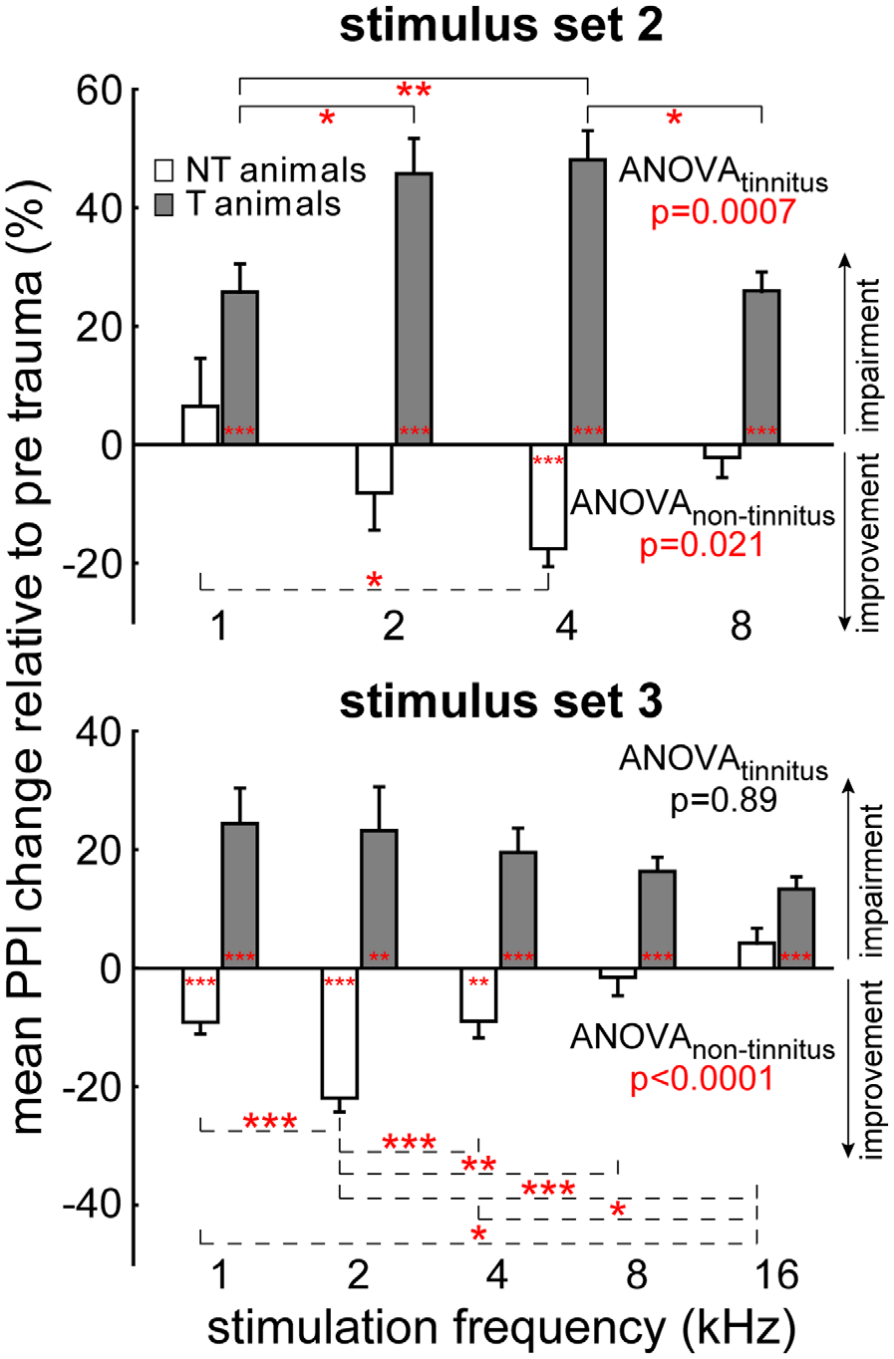
Behavioral tinnitus tests (gap-noise paradigms) in 35 animals. Mean normalized PPI response changes relative to pre trauma (in percent ± SEM) of group T (black) and NT (white); upward deviations indicate PPI impairment indicative for an existing tinnitus percept, downward gives PPI improvement. Upper panel: stimulus set 2, the responses are sorted for startle stimulus frequency. Lower panel: stimulus set 3, the responses are sorted for the bandpass noise center frequency. Significance levels of single sample t-tests vs. 0 indicating an impairment or improvement for each frequency and the Tukey-tests of the 1-factorial ANOVA indicating frequency specific impairment or improvement: * p<0.05, ** p<0.01, *** p<0.001.

Figure 3 compares the results of the two behavioral paradigms for tinnitus detection. The top panel shows the behavioral data for stimulus set 2 across all animals, the bottom panel depicts data for stimulus set 3. Grey bars show post trauma PPI changes in animals that developed a tinnitus percept (group T), white bars those for animals that did not develop a tinnitus (group NT). For group T (narrow and wide tinnitus percept) and stimulation set 2 (Figure 3, top panel) a significant impairment of PPI as a sign of tinnitus could be detected at all tested frequencies after the trauma (single sample t-tests vs. 0 all p<0.001; mean ± SD at 1 kHz: 25.7±95.0%; 2 kHz: 45.7±114.7%; 4 kHz: 48.1±98.3%; 8 kHz: 25.9±49.1%) but was strongest at 2 and 4 kHz (Tukey-tests after significant one-factorial ANOVA). NT animals on the other hand showed no impairment or even a significant improvement of PPI (1 kHz: 6.5±93.0%; 2 kHz: −8.2±72.0%; 4 kHz: −17.6±35.4%, p<0.001; 8 kHz: −2.2±32.8%). In stimulation set 3 (Figure 3, bottom panel) T animals showed a significant impairment of PPI at all tested frequencies after the trauma (single sample t-tests vs. 0 all p<0.001 except 2 kHz where p = 0.002; 1 kHz: 24.5±126.9%; 2 kHz: 23.2±158.1%; 4 kHz: 19.6±86.4%; 8 kHz: 16.3±24.8%; 16 kHz: 13.4±19.8%) while the distribution – without any clear peak as indicated by the non-significant one-factorial ANOVA – was not identical to the one shown above (Kolmogorov-Smirnov test, p<0.05). NT animals again showed no impairment or even improved their PPI responses significantly (1 kHz: −9.1±23.8%, p<0.001; 2 kHz: −21.9±28.7%, p<0.001; 4 kHz: −8.9±33.5%, p = 0.002; 8 kHz: −1.5±14.9%; 16 kHz: 3.7±13.3%). In other words, whereas group T developed significantly impaired PPI as a sign of tinnitus in both paradigms, NT animals even showed significantly improved PPI at frequencies from one octave below to one octave above the trauma frequency compared to pre trauma conditions.

**Figure 4:**
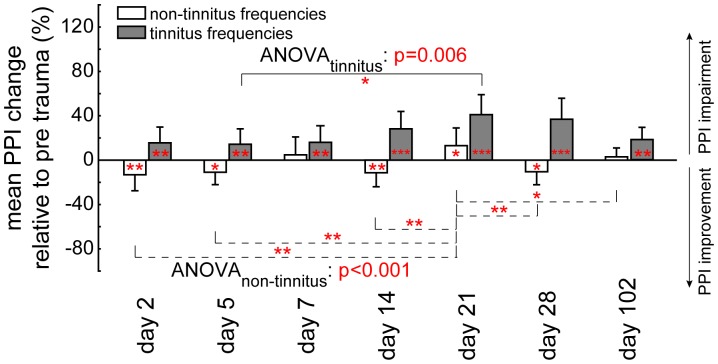
Development of tinnitus percept over time (stimulation set 2). Mean prepulse inhibition change relative to pre trauma response (in percent ± SEM) for non-tinnitus frequencies (open bars) and all tinnitus frequencies (black bars) obtained up to 102 days after trauma, exemplary in four T animals. An impairment of PPI is plotted upward, an improvement downward; significant difference of data to pre trauma is depicted by asterisks in each bar (one sample t-tests vs. 0). Connecting lines indicate significant Tukey post-hoc tests. Significance levels: * p<0.05, ** p<0.01, *** p<0.001.

In Figure 4 the development of the tinnitus percept is exemplarily assessed over time in 4 T animals by 1-factorial ANOVAs in tinnitus and non-tinnitus frequencies (defined at day 2 post trauma) separately. The impairment of PPI, as indicative of a tinnitus percept in these T animals increased over time and reached a peak after 3 to 4 weeks post trauma. The data demonstrate (t-tests vs. 0) that the acute tinnitus percept is evident from day 2 post trauma on. At 3 weeks post trauma we also find the non-tinnitus frequencies to be affected; this may point to a peak tinnitus percept around that time which than becomes chronic (tested up to 16 weeks) and the non-tinnitus frequencies become unaffected again. Therefore, the neuro-plastic processes that lead to the development of a chronic subjective tinnitus seem to be finished after about 3 weeks post trauma in this animal model.

**Figure 5:**
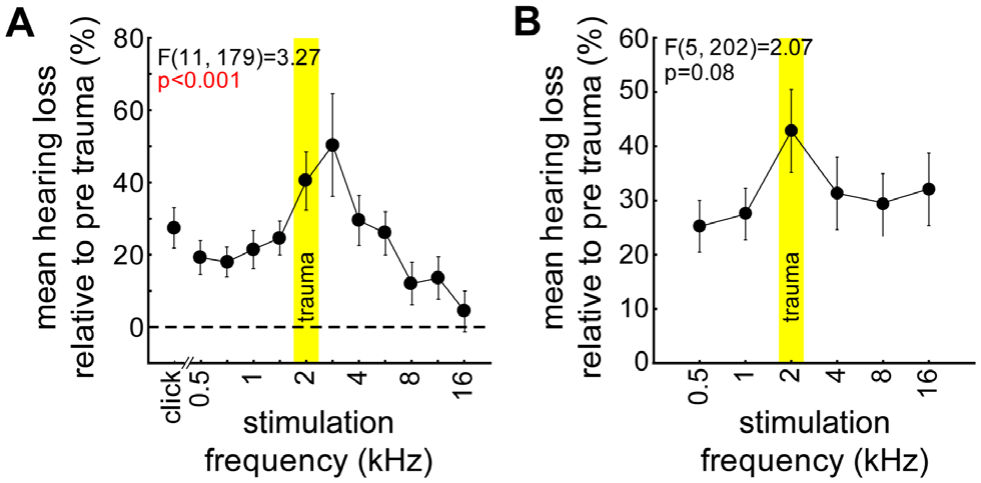
ABR and behaviorally determined hearing loss. **A** Significant one-factorial ANOVA (mean ± SEM) of the hearing loss (in percent relative to pre trauma) over the different stimulation frequencies as obtained by ABR measurements of all investigated animals. The yellow area indicates the trauma frequency. **B** One-factorial ANOVA of the hearing loss obtained by behavioral threshold paradigm (stimulus set 1). The statistics indicate only a tendency for frequency specific hearing loss around the trauma frequency. In both methods of HL determination the trauma frequency is affected most.

All animals showed a significant hearing loss (HL) which was similar in their ABR and behavioral audiograms (Figure 5). The ABR thresholds averaged over all animals and frequencies increased significantly: mean ABR after the trauma pre 44.2±11.2 dB SPL, post 52.9±11.6 dB SPL (F (1, 732) = 153.51, p<0.001). As can be seen in Figure 5A this HL was frequency dependent, as indicated by the significant one-factorial ANOVA for the HL over all stimuli (click and pure tones of different frequencies; F(11, 179) = 3.27; p<0.001) with a maximal HL around the trauma frequency and half an octave above. The HL assessed by the behavioral audiogram is depicted in Figure 5B. We also find a general threshold increase (single sample t-tests HL for each frequency vs. 0, always p<0.001) but the one-factorial ANOVA only shows a tendency for frequency-specificity (F(5, 202) = 2.07, p = 0.08).

We also analyzed the HL of both tinnitus animal groups (T and NT) dependent on the stimulation frequency by 2-factorial ANOVAs and found neither in the ABR nor in the behaviorally determined thresholds any frequency specific differences between the animal groups. This was true for the interactions as well as for the mean HL over all frequencies which in ABR measurements of the NT animals amounted 23.2±23.8% and in the T animals 24.0±30.5% (2-factorial ANOVA, F(1, 167) = 0.03; p = 0.87) and 70.4±49.2% and 82.8±51.9% (2-factorial ANOVA, F(1, 162) = 1.76, p = 0.19) in the behavioral audiograms for NT and T animals, respectively. In other words, the existence or non-existence of a tinnitus percept in our animal groups cannot be explained by differences in the induced trauma as determined with these physiological or behavioral measures. Nevertheless, as the variance between the different specimens was quite high in both groups (large standard deviations) an effect on the individual HL level cannot completely be ruled out.

### Neurophysiological Correlates of HL and Tinnitus in Primary Auditory Cortex Field AI

The behavioral differences in T and NT animals were paralleled by different plastic changes of neuronal responses in primary auditory cortex field AI: We recorded pure tone responses from a total of 627 single and multi-units (490 units in T and 137 units in NT group). Out of these, 331 units were recorded before (278 in T, 53 in NT group) and 296 units after the acoustic trauma (212 in T, 84 in NT group) over several recording sessions, usually 2 sessions before and 3 after the trauma.

**Figure 6:**
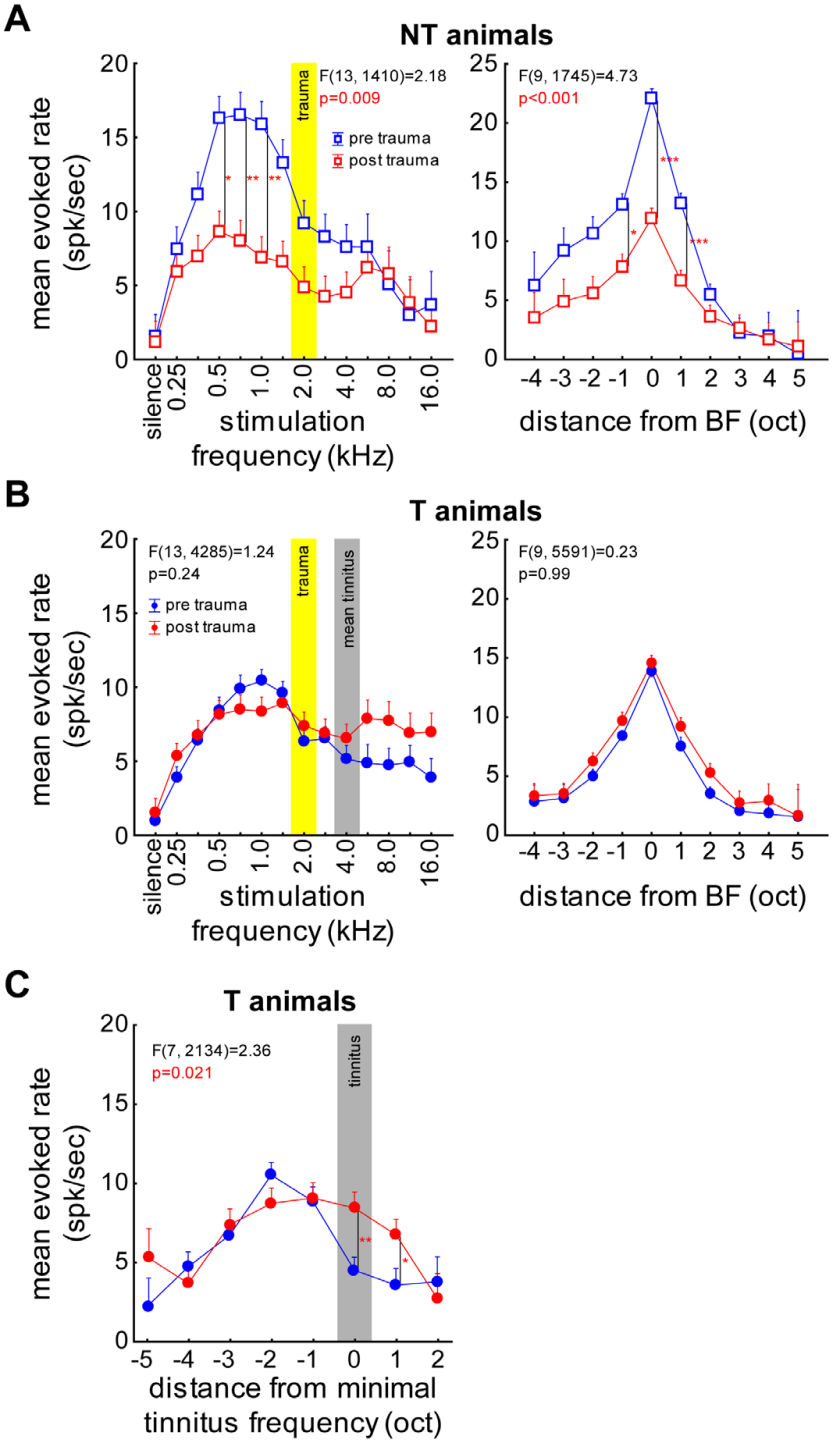
Neuronal evoked response to iso-intensity pure tone stimulation. Mean response (+SEM) of all recorded neurons of groups NT and T during 70 dB SPL stimulation. **A** **left panel** Shown is the interaction of the 2-factorial ANOVA of trauma status (blue: before; red: after) over frequencies (trauma: yellow area) of the pure tone evoked response rates in group NT. **right panel** Interaction of the 2-factorial ANOVA of trauma status and frequencies (octaves) of mean response rates aligned to the BF of each individual unit. NT animals show a strong decrease in evoked rate below 2 kHz and around the BF after the trauma. **B** **left panel** Same display as in A for group T with the mean minimal tinnitus frequency (grey bar). **right panel** Same display as in A, data aligned to the BF of each unit. Note that in this group the BF shifts after the trauma towards higher frequencies (cf. panel C). T animals do not show this reduction of evoked rate that NT animals exhibit. **C** Interaction of frequency (octaves) and trauma status of data from group T aligned to individual minimal tinnitus frequency now becomes significant with increased mean evoked rate after the trauma at the tinnitus frequency and one octave above. T animals show a frequency specific increase of evoked response after the trauma with aligning the recorded data to the behaviorally determined tinnitus frequency. Tukey post-hoc test significance level: * p<0.05, ** p<0.01, *** p<0.001.

Figure 6A shows the mean evoked response rates as a function of pure tone frequency across all units in NT animals before and after trauma (left panel). The same data were replotted in the right panel but now aligned to the BF of each unit. With a 2-factorial ANOVA we found an overall decrease of evoked rate in NT animals after the trauma (pre: 11.50±1.07 spikes/sec, post: 6.14±0.53 spikes/sec, F(1, 1410) = 43.98, p<0.001) that was not uniformly distributed across all frequencies (2-factorial ANOVA; p = 0.009) but most prominent below the trauma frequency (significant Tukey-tests at 0.5, 0.7 and 1 kHz). Figure 6B depicts the respective mean evoked rates recorded in the T animals. No significant change of mean rate averaged over all frequencies (pre: 7.11±0.37 spikes/sec, post: 7.88±0.61 spikes/sec, F(1, 4285) = 2.04, n.s.) nor any interaction between frequency and trauma status could be found. Interestingly, comparing the data from Figure 6A and 6B, it is obvious that the mean evoked rates in T and NT animals were different before the trauma (mean over all frequencies for NT: 10.88±15.14, T: 6.75±10.59; F(1, 4260) = 83.43, p<0.001; interaction: F(21, 4260) = 2.49, p<0.001, Tukey-tests significant at 0.5, 0.7 and 1 kHz) but reached similar levels after the trauma (no significant difference over all frequencies for NT: 6.82±8.21 and T: 7.49±14.78, F(1, 3526) = 1.17 or in interaction of frequency and group F(21, 3526) = 0.40; p>0.05 for both). The mean evoked response rates in group T showed a significant increase only when aligning the data to the individually perceived lowest tinnitus frequency (Figure 6C). Tukey post-hoc tests indicated these differences to be exactly at this tinnitus frequency and one octave above and therefore this may be a neuronal correlate of the tinnitus percept.

**Figure 7:**
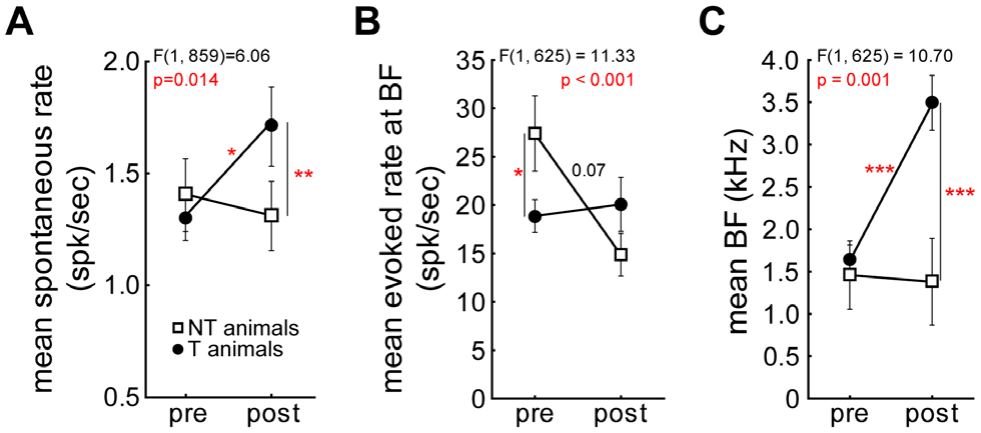
Trauma induced changes of neuronal response parameters. Interactions of the ANOVAs of the mean values (± SEM) of neuronal response parameters in groups T (filled circles) and NT (open squares) before and after acoustic trauma. **A** Significant interaction of trauma status and animal group in the spontaneous rate with an increase in activity after the trauma in T animals only. **B** Evoked rate at BF is different before but not after the trauma between both groups. **C** BF of all recorded units changes only in T animals after the trauma indicating a reorganization of tonotopic maps. Tukey post-hoc test significance levels: * p<0.05, *** p<0.001.

Further changes or predispositions of neuronal response properties were observed as summarized in Figure 7. First of all, the described changes in evoked discharge rates were “anti-paralleled” by changes in the spontaneous rate that significantly increased in the T group whereas it did not change in the NT group (Figure 7A) after the trauma. These changes or non-changes in spontaneous rate may also represent neuronal correlates for the existence or non-existence of a tinnitus percept in group T or NT, respectively. Figure 7B demonstrates that the pure tone evoked response rate at the BF was different between groups T and NT already *before* the trauma, which could already be inferred from the analyses described in Figure 6. Furthermore, in line with previous reports [5], [7] we observed a significant change in mean BF in the T group that is indicative for a neuro-plastic change in the functional topography of the tonotopic organization of AI that is not seen in the NT group (Figure 7C).

**Figure 8:**
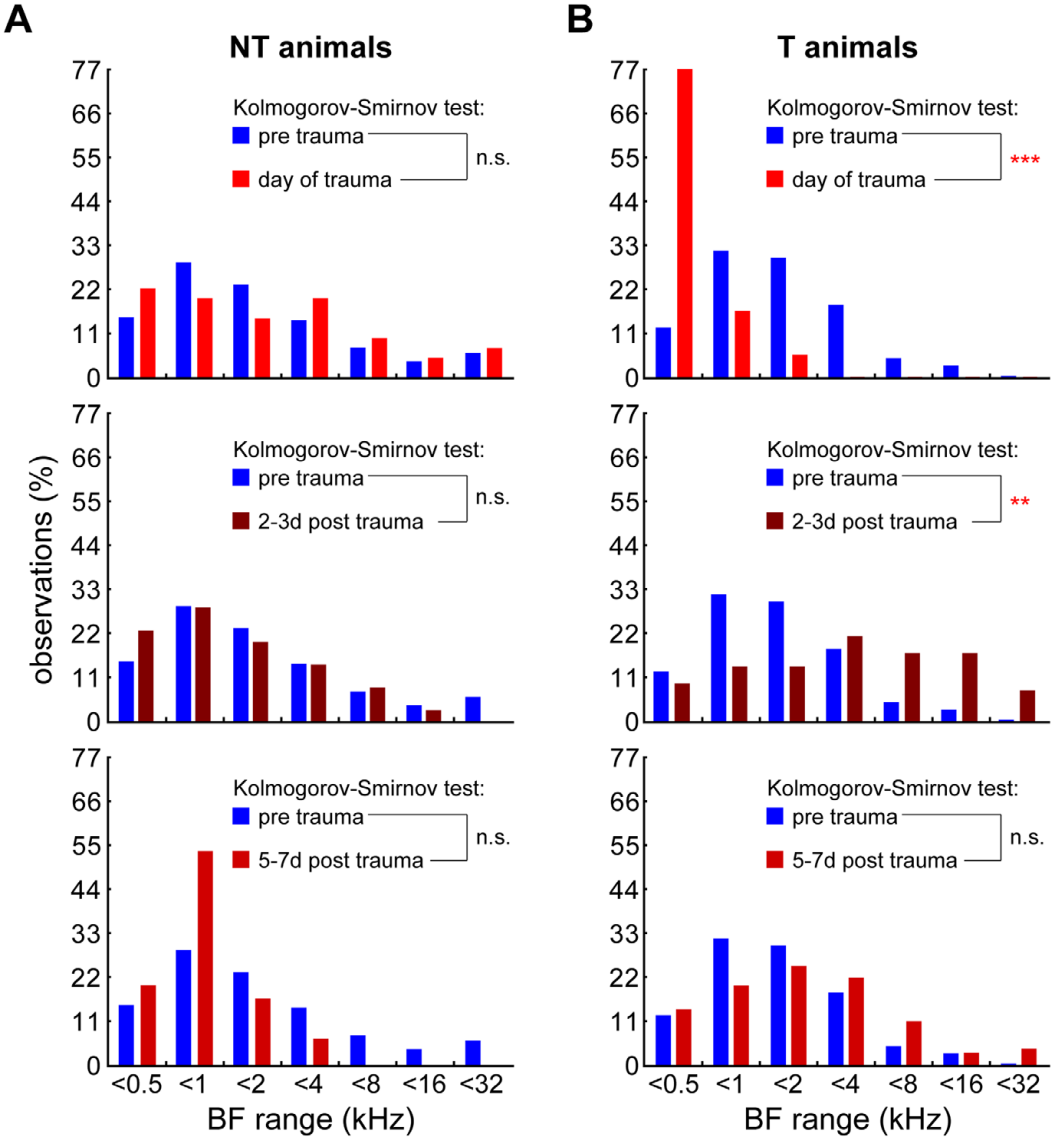
Changes in BF frequency distributions over time. A Comparison of the frequency distribution of BF (observations in %) binned in one octave steps of NT animals before the trauma (blue) with the data obtained on 3 different time points post trauma, from top to bottom: day of trauma, 2 to 3 days after trauma, 5 to 7 days after trauma. Distributions are tested by Kolmogorov-Smirnov tests corrected for multiple comparisons. The distributions do not change over time. **B** Comparison of the BF distributions of T animals on the same three time points. The distributions show strong shifts over time. Kolmogorov-Smirnov significance levels: ** p<0.01, *** p<0.001.

This trauma-induced plasticity of the tonotopic organization in AI of T animals was further analyzed and compared to NT animals in Figure 8: There, the spatio-temporal dynamics of the changes are plotted as a function of time and BF for both T and NT animals (right and left column, respectively).,We separated the data for the day of recording into four groups, first, the pre trauma data (blue) and second the recordings performed at three different time ranges post trauma (reddish colors), namely the day of the trauma, i.e., immediately after obtaining the ABR (top panels), day 2 to 3 post trauma and finally days 5 to 7 post trauma. The BFs of units in these groups were binned in octave bands and the frequency distributions of the BFs of T and NT animals at the three post trauma time points were compared to the pre trauma condition (tested with Kolmogorov-Smirnov tests, corrected for multiple testing). As can be seen in Figure 8, the BF distributions of NT animals (Figure 8A) did not show any significant changes over time. On the other hand, the BF distributions of the T animals (Figure 8B) – while not different from NT animals before the trauma – did show strong and significant shifts over time: On the day of the trauma we found a strong shift to an over-representation of frequencies below the acoustic trauma, on day 2 and 3 the whole distribution shifted to an over-representation of frequencies above the trauma frequency, and finally after one week the distribution changed back to pre trauma conditions. In other words, after dramatic disturbances of the tonotopic organization immediately post trauma, the animals showing a tinnitus percept, seem to be completely normal again in their tonotopic organization of AI by one week post trauma, although the tinnitus percept was still present at that time (cf. Figures 3 and 4).

**Figure 9:**
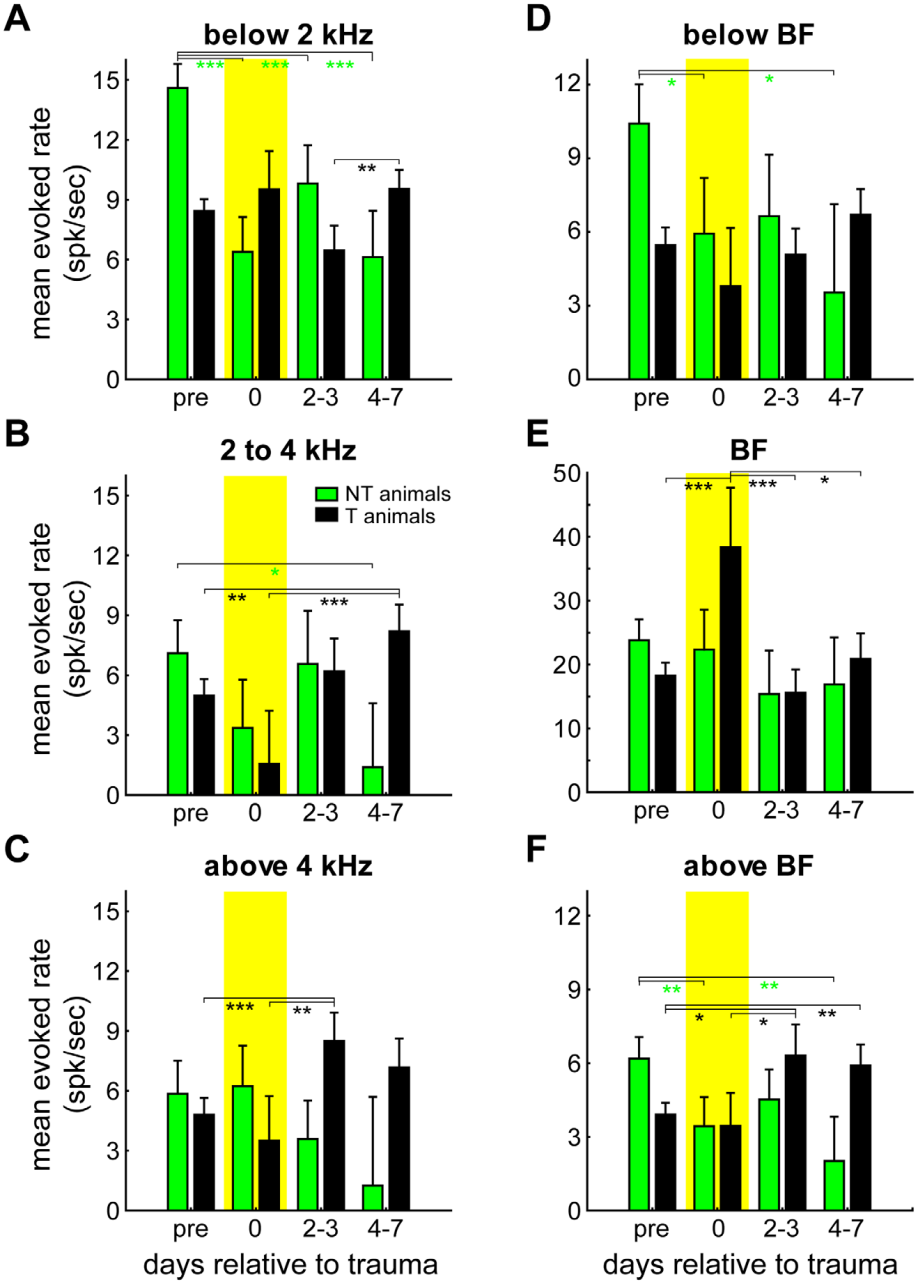
Changes in mean evoked rate over time. Results of the 2-factorial ANOVAs of mean evoked rates of all recorded units as a function of recording time and animal group (green = NT, black = T) separately for different frequency ranges; all interactions are significant with p<0.001, asterisks indicate significant Tukey post-hoc tests, whiskers give the SEM. **A** Mean evoked rate of all units stimulated with frequencies below the trauma; only NT animals show a decrease in responses relative to pre trauma. **B** Mean evoked rate at trauma affected frequencies; increase of response over time only in T animals. **C** Mean evoked rate above the trauma frequencies; again, only T animals show increase of response rates over time. **D** Mean evoked rate of frequencies at least 1 octave below the individual BF of every unit; only NT animals show decrease of activity relative to pre trauma. **E** BF responses of all recorded units; in T animals a significant increase of response strength can be found only at the day of the trauma, immediately post trauma. **F** Mean evoked rates at least one octave above BF; in T animals an increase of response can be found only from day 2–3 on, while in NT animals the responses decrease already at the day of the trauma. Tukey post-hoc test significance levels: * p<0.05, ** p<0.01, *** p<0.001.

Because of this finding of these temporal dynamics of the noise-trauma induced plasticity of the tonotopic organization, we also analyzed the temporal dynamics of the rate changes shown in Figure 7A and B: While the T animals showed an almost linear increase of the spontaneous rate over time, no changes of spontaneous rate over time were seen in the NT group (not shown). In contrast, changes in evoked discharge rate showed complex temporal dynamics over time: Figure 9A to F give the interactions in the 2-factorial ANOVAs of the mean evoked rates for the same 4 time ranges depicted above (cf. Figure 8) relative to trauma induction (pre trauma, immediately after trauma, 2 to 3 days post trauma and 5 to 7 days post trauma) in both animal groups; all interactions are significant at a p-level below 0.001 allowing Tukey post-hoc tests. Shown are mean evoked rates across all units in AI stimulated with frequencies below the trauma frequency (Figure 9A), at the trauma affected frequencies (2 to 4 kHz; Figure 9B, cf. Figure 5A) and frequencies above the trauma (Figure 9C). As can be seen, the different animal groups show different temporal dynamics of evoked rate changes within these three frequency ranges: Whereas NT animals show a strong and significant decrease of evoked rate below the trauma frequency immediately after the trauma with no further significant changes during the following week (NT_pre_ vs. NT_0_ to NT_4–7_: Tukey post-hoc tests always p<0.001), T animals display no significant changes of mean evoked rate with low frequency stimulation relative to the pre trauma status with only one minor fluctuation (T_2–3_ vs. T_4–7_: p = 0.003, Figure 9A). In contrast, at and above the trauma frequency (Figure 9B, C), NT animals show less changes with strongest decrease of mean evoked rate towards one week post trauma at the trauma frequency range (NT_pre_ vs. NT_4–7_: p = 0.04), while the T animals show a significant increase of the mean evoked rate by that time at the frequencies where the tinnitus is perceived (trauma frequencies: T_pre_ vs. T_4–7_: p = 0.001, T_0_ vs. T_4–7_: p<0.001; above trauma frequencies: T_pre_ vs. T _2–3:_ p<0.001, T_0_ vs. T_2–3_: p = 0.005).

Interestingly, when mean evoked rate changes are plotted only for responses at the BFs of the individual units, a different picture was found (Figure 9E): There, no significant changes could be detected for the NT group while within the T group there was a strong significant increase of mean BF-evoked rate immediately after the trauma that returned back to normal on day 2 to 3 post trauma (T_0_ vs. T_pre_ and T_2–3_: p<0.001, T_0_ vs. T_4–7_: p = 0.02). For stimulation frequencies at least one octave below BF, no significant changes were seen for T animals while there was a decrease in response rate for NT animals (NT_pre_ vs. NT_0_: p = 0.03, NT_pre_ vs. NT_4–7_: p = 0.01, Figure 9D), and for frequencies at least one octave above BF (Figure 9F) there was a significant increase in mean response rate in T animals, but this change was only seen after the changes at BF had vanished again, i.e., from day 2–3 on (T_pre_ vs. T_2–3_: p = 0.01, T_pre_ vs. T_4–7_: p = 0.002, T_0_ vs. T_2–3_: p = 0.04). For the NT animals, again a decrease in response rate over time was seen at this frequency range (NT_pre_ vs. NT_0_: p = 0.006, NT_pre_ vs. NT_4–7_: p = 0.001).

**Figure 10:**
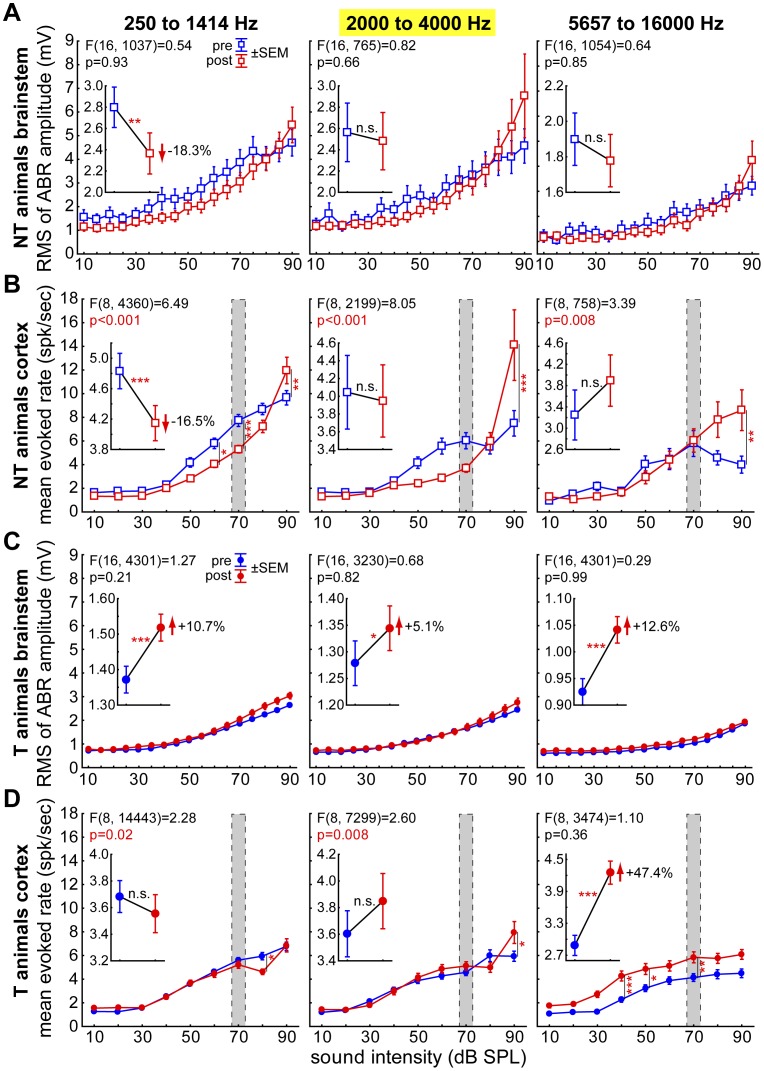
Intensity functions of brainstem and cortical responses in NT and T animals. A Brainstem responses (RMS of ABR amplitudes) of NT animals over the different stimulation intensities grouped for frequencies below the trauma, frequencies affected most by the trauma and frequencies above the trauma affected range. Given is the interaction of the 2-factorial ANOVA of measurement time (pre or post trauma) and intensities, Whiskers give the SEM, asterisks indicate significant Tukey post-hoc tests. No interaction is found here while the mean over all intensities (insets) indicates a significant decrease of ABR response after the trauma for frequencies below 2 kHz. **B** Mean rate-intensity functions of the evoked responses of the neurons in AI in NT animals plotted the same way as in A, the gray area indicates the iso-intensity measurement range shown, e.g., in Figure 6. The interaction is significant in all three cases indicating intensity specific changes while over all intensities only the activity below the trauma drops significantly. **C** ABR of T animals, no significant interaction could be found, but mean over all intensities the ABR amplitudes increase in all frequency ranges. **D** Mean rate-intensity functions of neurons in AI of T animals, the interaction is significant for the lower two frequency ranges indicating intensity specific changes without a general increase of activity. This can only be found above the trauma in the range of the tinnitus frequencies. Tukey post-hoc test significance level: * p<0.05, ** p<0.01, *** p<0.001.

Figure 10 finally depicts mean ABR strength (root-mean-square (RMS) values of ABR amplitudes; A, C) and evoked rate (rate-intensity functions; B, D) as a function of sound intensity in T (C, D) and NT animals (A, B). We compared data measured before and directly after the trauma by 2-factorial ANOVAs for three frequency bands, namely below the trauma frequency (left column), the frequencies most affected by the trauma (2 to 4 kHz; middle column, cf. Figure 5A) and the frequencies above this affected range (right column).

Interestingly, the significant decrease in mean response rate of AI neurons of the NT group that was shown in Figure 6A could only be detected for moderate sound pressure levels (grey area in Figure 10B) and was only significant for frequencies below the trauma frequency (Figure 10B, left panel inset). For high sound pressure levels of 90 dB SPL, we found the inverse effect in group NT, namely a general increase in mean response rate after the trauma across all tested frequency ranges (Figure 10B). In contrast, no such changes were seen in the T group: There, we observed only minor changes in mean response rate in the rate-intensity functions for frequencies up to 4 kHz (Figure 10D, left and middle panels), but a strong and general increase of almost 50% across all intensities for frequencies above 4 kHz with a parallel shift of the whole mean rate-intensity function. In general, there was no such shift but rather a change in the shape of the rate-intensity functions in the NT animals (from a sigmoidal to an exponential curve progression; Figure 10B).

Comparing these changes of cortical rate-intensity functions to the ABR data we found that the observed changes of cortical response rates were in general paralleled by similar changes in ABR strength, with the exception that significantly strengthened responses in the T animals after trauma were seen in all frequency ranges and not only above 4 kHz (Figure 10C). Therefore it may be concluded that at least some of the changes we observed in AI simply reflect changes that occur already at levels of the auditory pathway downstream of the auditory cortex, although top-down influences are also conceivable (cf. [21], [22]).

Finally, comparing the overall level of mean ABR strength and evoked discharge rate between T and NT animals (Fig. 10 A vs. C and B vs. D, respectively), we found generally smaller ABR amplitudes (F(1, 14888) = 1296.7, p<0.001) and the lower spike rates in AI (F(1, 32637) = 65.57, p<0.001) before and after trauma in the T compared to the NT animals.

## Discussion

### Methodological Considerations

In this report we aimed to understand why different individuals suffering from similar peripheral auditory impairment often but not always do develop a tinnitus percept. Therefore it is crucial to this study to undoubtedly identify those animals that did develop a tinnitus percept after noise trauma and distinguish them from those who did not. To achieve this goal we employed a multistep procedure: First, we quantified the noise trauma induced hearing loss using both electrophysiological (ABR) and behavioral approaches (PPI of ASR) which led to similar estimates of hearing loss (cf. Figure 5; [16]). Only those animals were included in the study that showed a hearing impairment of at least 15 dB at the trauma frequency of 2 kHz to prevent any effect of hidden hearing loss [23]. Thereby, the paradigm we used to induce this hearing loss was relatively mild, which in recent studies turned out to be ideal to induce the development of tinnitus in rodent models compared to severe acoustic traumata, probably because in the latter case the hearing loss is less restricted to a certain frequency range and therefore the effect on decreased lateral inhibition is spectrally less focused. [24], [25]. Second, we used two different behavioral approaches to detect a tinnitus percept in our animals, namely the gap-noise modulated PPI paradigm adapted from Turner and colleagues [12], [20], [26] and fear-potentiation modulated PPI paradigms, e.g., inspired from Guitton and colleagues [15]. Although the two methods yield slightly different estimates of tinnitus frequency (cf. Figure 3), the outcome was highly comparable with respect to the question if there was a tinnitus percept at all or not as all animals displayed a tinnitus percept in both paradigms with at least one overlapping frequency. We normalized all PPI responses individually for all frequencies tested to counteract the effects of the different perception thresholds and of the trauma. We then grouped the animals according to their behavior into individuals with and without tinnitus and investigated the pure tone responses of neurons within AI. Finally, we correlated the neurophysiological data (ABR and AI recordings) with the behavioral data. The fact that we could describe a number of highly significant differences in the neuronal responses pre and post trauma between animals classified behaviorally as having a tinnitus percept (group T) or not (group NT) further strengthens the results of the behavioral tinnitus detection procedures employed here (cf. Figures 6 to 10). Therefore, based on this combination of behavioral and electrophysiological parameters collected in this report it is very likely that the individual specimen tested here were correctly grouped into T and NT animals.

### Predisposition for Subjective Tinnitus Development

The most prominent pre trauma differences in T and NT animals were the higher sound-evoked activities within the auditory system (as apparent in both ABR and AI recordings) of the NT group compared to the T group (cf. Figures 6; 7B; 9 and 10). Obviously this higher neuronal activity allows the auditory system of animals in the NT group to differently react to noise-induced peripheral damage compared to the T animals. One might speculate on the different neuronal mechanisms that lead, after a noise trauma, to the development or prevention of tinnitus in T and NT animals, respectively:

In T animals the trauma-induced damage to the receptor epithelium of the cochlea obviously triggers a number of neuroplastic changes throughout the auditory system that may be either transient or permanent (cf. [27], [28]). In our model, the plastic reorganization of the tonotopic organization of the primary auditory cortex that has already been described by a number of studies [29], [30], [31], [32], [33], [34] turned out to be only transient, but showed complex temporal dynamics: A shift of the BF-representation to lower frequencies immediately after trauma was followed by a shift to higher frequencies a few days later and back to normal after about one week post trauma (cf. Figure 8). Intriguingly, these changes in tonotopic organization in AI were accompanied by significantly but also transiently increased response rates at the BF of the units (Figure 9E). As changes at off-BF frequencies appeared later and were permanent rather that transient (Figure 9F), this points to a mechanism of plastic changes that affect the tonotopic organization and which are active within the receptive field of the units during a short, transient post trauma period. During this temporal disturbance of the tonotopic organization in AI obviously some further plastic changes take place that stay permanent even after the tonotopic order is back to pre trauma conditions. These seem to be most prominent at frequencies above BF, i.e. above the center of the spectral receptive field. In our data, these plastic changes are represented by a number of increased (spontaneous and evoked) neuronal response rates that are in part stimulation frequency specific and correlated to the behaviorally estimated perceived tinnitus frequencies (cf. Figures 6C; 7A; 9C, F; 10C, D). We believe that these neurophysiological changes described during the first week post trauma reflect the transition from an acute to a chronic state of subjective tinnitus in the T group, as the tinnitus percept is still present after the plastic reorganizations are finished (cf. Figure 4). Finally, as the changes of the tonotopic representation in AI and evoked rates at BF were transient in the T animals whereas the increases in neuronal discharge rate – spontaneous and at high, tinnitus-related frequencies – persisted beyond one week post trauma, we believe that the latter are the neurophysiological correlates of the tinnitus percept rather than the former.

### Tinnitus Prevention

In NT animals we saw a different picture, as the noise-trauma induced neuroplastic changes were completely different compared to the T group: In the NT group, coming from a higher overall level of sound induced activity in the auditory system, we measured a significant decrease in evoked response rates both in AI and ABR recordings (cf. Figures 6A; 7B) but no change in spontaneous rate (cf. Figure 7A) or tonotopic organization (cf. Figures 7C; 8A). Interestingly it turned out that this reducing effect on evoked response rate could only be seen at moderate sound intensities, while there were no changes for low intensities and even increased evoked responses for the highest intensity testes (90 dB SPL; cf. Figure 10A, B). Based on these observations we propose the following model of tinnitus prevention in NT animals:

We believe that there is an active neuronal process that is able to prevent the development of a tinnitus percept in the NT group, whereas it is not in the T group. Based on our data the main predisposition for this ability to prevent tinnitus development seems to be the higher overall neuronal activity – both spontaneous and evoked – in the auditory system of the NT compared to the T animals. Obviously this neuronal process is able to reduce evoked activity, thereby preventing changes in spontaneous activity, evoked activity at high stimulation frequencies and tonotopic organization. A candidate for a transmitter system that may mediate this effect is the GABA_A_-system, as it allows for a fast and global inhibition of the whole auditory cortex in response to sound [35], [36], [37], [38], [39], [40], [41]. We believe that the inhibition relevant here is global rather than frequency specific, as the reduction in response rate is rather focused on frequencies below the trauma frequency (cf. Figures 6A; 9A, D; 10A, B), whereas the tinnitus percept seems to be related to increased response rates at frequencies above the trauma frequency (cf. Figure 6B; 9C, F; 10D). Our hypothesis is that a global inhibitory mechanism that counteracts the development of increased rates at high frequencies is able to prevent tinnitus development, but at the same time, as it acts in a non-frequency specific manner, reduces the response rates at lower frequencies. This mechanism seems to work only for moderate sound intensities that are in the range of the normal auditory surround of the animals, possibly because the stimulation of the auditory system in this intensity range is needed to further trigger the malfunctional development of tinnitus during a critical period post trauma. At lower intensities it may not be necessary to activate this global inhibitory mechanism to prevent the tinnitus development, and at high intensities, were we observed increased response rates even in the NT group, it may be overstrained so that tinnitus would still develop. As the latter possibility would be a situation of permanent ongoing noise exposure for the animals, it may well be that our NT animals would have developed a tinnitus if the loud stimulation would have continued permanently (continuous noise pollution) rather than only during short, 200 ms stimuli during recording. Both scenarios, noise reduced environments for T animals and noise-intense environments for NT animals after the trauma may be used to test these hypotheses in future studies.

We propose that the high pre trauma neuronal activity in the NT group allows for its described significant reduction, whereas the low pre trauma activity in the T group is not sufficient to trigger this mechanism or, alternatively, global cortical inhibition is already at its limits, causal for the lower neuronal activity in these animals and cannot be increased further. In this case, the plastic recalibrations of neuronal response rates that take place in the T animals in the course of tinnitus chronification also trigger disturbances of the tonotopic organization in AI that remain transient, probably because they merely reflect the response rate recalibration process itself [28], [42] and are again dominated by the tonotopic organization of the thalamic input after the rate recalibration is finished.

Finally, our view of an insufficient additional inhibitory capacity in T compared to NT animals is supported by recently published results from Yang and colleagues [43] reporting an abolishment of the tinnitus percept in rats after injection of GABAergic enhancers but not after applying excitation reducing pharmaceutics.

In summary we believe that an overall high neuronal activity in the auditory system opens the possibility to activate a global inhibitory mechanism that is able to prevent the development of a subjective tinnitus after a trauma-induced damage to the peripheral receptor epithelium of the cochlea. A closer understanding of this mechanism might open the possibility to develop a prophylaxis strategy to prevent the development of a subjective tinnitus in hearing impaired patients, e.g. after acute noise trauma. In this context, GABAergic enhancers [43] or other therapeutical interventions [10], [44] that are able to reduce overall neuronal activity may be a promising strategy to follow.
